# Evaluation of probiotics in the treatment of hypothyroidism in early pregnancy combined with small intestinal bacterial overgrowth

**DOI:** 10.1002/fsn3.3948

**Published:** 2024-01-22

**Authors:** Miao Zhang, Yajuan Xu, Zongzong Sun, Yanjie Ban, Shanshan Zhai, Wentao Wang, Mengqi Wang, Jie You, Dongsun Chen, Shuanghui Zhu, Hui Guo

**Affiliations:** ^1^ Department of Obstetrics and Gynecology The Third Affiliated Hospital of Zhengzhou University Zhengzhou China

**Keywords:** hydrogen/methane lactose breath test, hypothyroidism in pregnancy, probiotics, small intestinal bacterial overgrowth

## Abstract

The aim of this study was to investigate the association between hypothyroidism in early pregnancy and small intestinal bacterial overgrowth (SIBO) and the effect of probiotics. Patients with hypothyroidism in early pregnancy and normal pregnant women during the same period were included in the methane–hydrogen breath test to compare the incidence of SIBO, smoothed curve fit, and differences in clinical symptoms. For those who combined with SIBO, the rate of clinical symptom conversion, thyroid hormones, and changes in associated inflammatory indexes were compared after 21 days of treatment with probiotics on top of conventional levothyroxine sodium tablets. The results are as follows: (1) The incidence of combined SIBO in patients with hypothyroidism in pregnancy was 56.0%, significantly higher than the 28.0% of normal pregnant women during the same period. (2) The highest value of hydrogen plus methane gas in 90 min in pregnancy hypothyroid patients showed a significant negative correlation with FT4 (*p* < .001, SD = 0.169). (3) Abdominal distension symptoms were significantly increased in both groups after combined SIBO (*p* = .036, *p* = .025), and the conversion rate after treatment was 69.2% and 75.0%, respectively. (4) In hypothyroidism, pregnancy combined with SIBO, TSH, and CRP was higher before treatment (*p* = .001, *p* = .012) and decreased significantly after treatment (*p* = .001, *p* = .008). Hypothyroidism in early pregnancy is associated with SIBO, and probiotic treatment is significantly effective.

## Graphical abstract

Hypothyroidism in early pregnancy is associated with small intestinal bacterial overgrowth, which can lead to abdominal distension symptoms, and small intestinal bacterial overgrowth may be involved in the regulation of thyroid function. And probiotics have a significant effect in treating hypothyroidism in early pregnancy combined with small intestinal bacterial overgrowth.

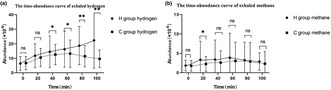

## 1 INTRODUCTION

Hypothyroidism in pregnancy has profound effects on maternal and child outcomes, especially in early pregnancy, and can directly lead to miscarriage, anemia, and so on (Moog et al., 2017; Thompson et al., 2018). Therefore, it is important to treat patients with hypothyroidism in early pregnancy. (Wang et al., 2021) found that the rate of positive small intestinal bacterial overgrowth (SIBO) was significantly higher in patients with subthyroidism during pregnancy than in controls. However, no definitive studies related to hypothyroidism and SIBO in pregnancy in early pregnancy have been reported.

Excessive growth of small intestine bacteria refers to the presence of excessive numbers of bacteria in the small bowel, which can cause gastrointestinal symptoms (Pimentel et al., 2020). Its clinical symptoms include malnutrition, abdominal distension, diarrhea, and abdominal pain (Chen et al., 2020; Xue et al., 2023) found that a quadruple probiotic combination consisting of *Bifidobacterium infantis*, *Lactobacillus acidophilus*, *Enterococcus faecalis*, and *Bacillus cereus* had anti‐inflammatory effects and could help repair damage of the intestinal barrier.

The aim of this study was to investigate the association between hypothyroidism in early pregnancy and SIBO and to preliminarily investigate the effect of probiotics on its treatment, which will hopefully be a new direction in the prevention and treatment of hypothyroidism in early pregnancy.

## 2 METHODS

### 2.1 Study subjects

Fifty cases of hypothyroidism in early pregnancy (<14 weeks) and 50 cases of normal pregnant women who met the criteria in our hospital from May to August 2022 were selected. All patients completed methane‐hydrogen breath test and tested for thyroid hormones and inflammatory indicators. The hypothyroidism in pregnancy group (H group) was divided into hypothyroidism in pregnancy combined with SIBO group (H‐S group) and hypothyroidism in pregnancy without SIBO group (H‐N group) according to the results of methane‐hydrogen breath test. Normal pregnant women (C group) were divided into normal pregnant women with combined SIBO group (C‐S group) and normal pregnant women without combined SIBO group (C‐N group). The study was approved by the Ethics Committee of local review board.

#### 2.1.1 Inclusion criteria

(1) According to the “Guideline on diagnosis and management of thyroid diseases during pregnancy and postpartum (2 edition)” and our hospital's laboratory, hypothyroidism in early pregnancy are eligible for FT4 < 12.3 pmol/L and TSH > 4.2 mIU/L. (2) Normal pregnant women need to have no pregnancy complications.

#### 2.1.2 Exclusion criteria

(1) Patients<18 years of age. (2) Patients with subclinical hypothyroidism, hyperthyroidism, gestational diabetes, and other pregnancy complications. (3) Patients receiving medication for hypothyroidism in pregnancy. (4) Patients with severe anxiety and depression. (5) Patients with a history of circulatory, digestive, and immune system disorders. (6) Patients who have been diagnosed with severe intestinal disorders or have undergone intestinal surgery. (7) Patients who have received antidiarrheal, probiotic, or antibacterial medications within the past 2 weeks. (8) Patients who take probiotic products daily.

### 2.2 Research methods and indicators

#### 2.2.1 The lactulose breath test

The experimental steps are as follows (Liu et al., 2016; Zhang et al., 2021): (1) 12 h before the test, all subjects were allowed to consume eggs, skinless chicken and fish, beef, and white rice. Dairy products, soy products, and other foods that tend to produce gas or are rich in fiber are prohibited; (2) on the day of the test, tooth brushing and mouth rinsing were required. Strenuous exercise were prohibited; (3) calibrate and test the levels of hydrogen and methane gas in the basal (fasting) breath. Subsequently, add 10 g of lactulose oral solution to 239 mL of warm water and allow the subjects to drink quickly. Exhaled breath was collected every 20 min. Time curves of hydrogen and methane gas abundance were plotted. The “breath analyzer Sc” used is manufactured by QuinTron.

SIBO can be diagnosed by meeting any of the following (Pimentel et al., 2020; Rezaie et al., 2017; Zhang et al., 2021): (1) hydrogen concentration is 20 ppm higher than the basal (fasting) value within 90 min; (2) methane gas concentration is 10 ppm higher than the basal (fasting) value within 90 min; (3) the hydrogen and methane gas concentrations did not reach the above values, but the sum was 15 ppm higher than the base value within 90 min.

#### 2.2.2 Diagnostic methods for gastrointestinal symptoms

The diagnostic criteria are as follows (Jani & Marsicano, 2018; Schiller et al., 2017; Zhang et al., 2021): (1) The diagnosis of abdominal distension needs to be met, in the past 3 months, repeated abdominal distension or obvious bloating for at least 3 days per month; (2) patients with increased frequency of bowel movements (more than 3 times per day) and changes in stool characteristics (pasty fluid) can be diagnosed with diarrhea; (3) The diagnosis of severe constipation includes two or more of the following conditions: (i) Sensation of straining during defecation more than one‐quarter (25%) of the time. (ii) The time when stool or stool becomes hard>25%. (iii) The time when there is a feeling of obstruction in the anus is>25%. (iv) The feeling of incomplete emptying>25% of the time. (v) Manual assistance with defecation >25% of the time. (vi) Defecation less than three times per week.

### 2.3 Treatment

According to “China's guidelines for prevention and management of thyroid diseases during pregnancy and perinatal period,” in patients with hypothyroidism in pregnancy, *levothyroxine sodium tablets* were given (2.0–2.4 μg/kg body weight per day), and no treatment was needed for normal pregnant women. While patients with combined SIBO in both groups were treated with *Combined Bifidobacterium*, *Lactobacillus*, *Enterococcus* and *Bacillus cereus* Tablets, Live (1.5 g 3 times a day), which were mainly composed of *Bifidobacterium infantis* (≥0.5 × 10^6^ CFU), *Lactobacillus acidophilus* (≥0.5 × 10^6^ CFU), *Enterococcus faecalis* (≥0.5 × 10^6^ CFU), and *Bacillus cereus* (≥0.5 × 10^5^ CFU). To compare the changes in clinical symptoms and SIBO conversion rate of patients in the group of hypothyroidism in pregnancy combined with SIBO and normal pregnant women combined with SIBO after 21 days of treatment. To compare the changes in thyroid hormone levels and related inflammatory indexes in the combined SIBO group and the uncomplicated SIBO group in hypothyroidism in pregnancy after 21 days of treatment. For those who do not turn negative for SIBO after 21 days of treatment, we will continue treatment.

### 2.4 Statistical analysis

Statistical analysis was performed using SPSS version 22.0. Data were expressed as frequencies and percentages. Comparisons between groups were made using the χ^2^ test, the corrected chi‐square test, or Fisher's exact probability test. Quantitative data with normal distribution were expressed as mean ± standard deviation (*x* ± *s*) and compared between groups using *t*‐test or ANOVA. Spearman's correlation analysis was used to analyze the association between the two variables. *p* < .05 was considered statistically significant. Smoothed curves were fitted using Empower Statistics software.

## 3 RESULTS

### 3.1 Comparison of general conditions

As shown in Table 1, FT4 and TSH were significantly higher in the hypothyroidism in pregnancy group than in the normal pregnant women group (*p <* .001, *p* < .001). Between the two groups, there was no statistically significant difference in age, height, weight, BMI, gestational week, gestation, and delivery.

**TABLE 1:** Comparison of general conditions.

	H group (*n* = 50)	C group (*n* = 50)	*p*
Mother's age (years, $\bar{\chi }$ ± S)	31.000 ± 4.924	30.880 ± 5.049	.904
Height (m, $\bar{\chi }$ ± S)	1.634 ± 0.052	1.616 ± 0.047	.067
Weight (kg, $\bar{\chi }$ ± S)	66.360 ± 6.067	64.62 ± 5.481	.136
BMI (kg/m^2^, $\bar{\chi }$ ± S)	25.840 ± 2.853	25.120 ± 1.637	.126
Week of pregnancy (weeks, $\bar{\chi }$ ± S)	25.800 ± 6.824	25.780 ± 5.527	.987
Number of pregnancies [times, M (P25, P75)]	1.50 (1.00, 2.00)	2.000 (1.00, 2.25)	.496
Number of births [times, M (P25, P75)]	0.00 (0.00, 1.00)	0.00 (0.00, 1.00)	.257
FT4 (pmol/L, $\bar{\chi }$ ± S)	9.120 ± 1.619	17.003 ± 2.726	<.001*
TSH (mIU/L, $\bar{\chi }$ ± S)	8.474 ± 4.092	2.110 ± 0.793	<.001*

*Note*: H group refers to the hypothyroidism in pregnancy group, C group refers to the normal pregnant women.

* In a row indicate that the medians of the different groups are significantly different (*p* < .05).

### 3.2 Experimental results of the lactulose breath test

The SIBO positivity rate was 56.0% and 28.0% in the hypothyroidism in pregnancy group and normal pregnant women group, respectively, and the difference was statistically significant (*p* = .005). Methane gas was consistent with SIBO positivity in 2.0% of the hypothyroidism in pregnancy group and 6.0% of the normal pregnant women group (*p* = .610). Hydrogen was consistent with SIBO positivity in 50.0% of the hypothyroidism in pregnancy group and 22.0% of the normal pregnant women group (*p* = .004). Both methane gas and hydrogen were consistent with SIBO positivity in 4.0% of the hypothyroidism in pregnancy group and 0.0% of the normal pregnant women group (*p* = .475).

**FIGURE 1:**
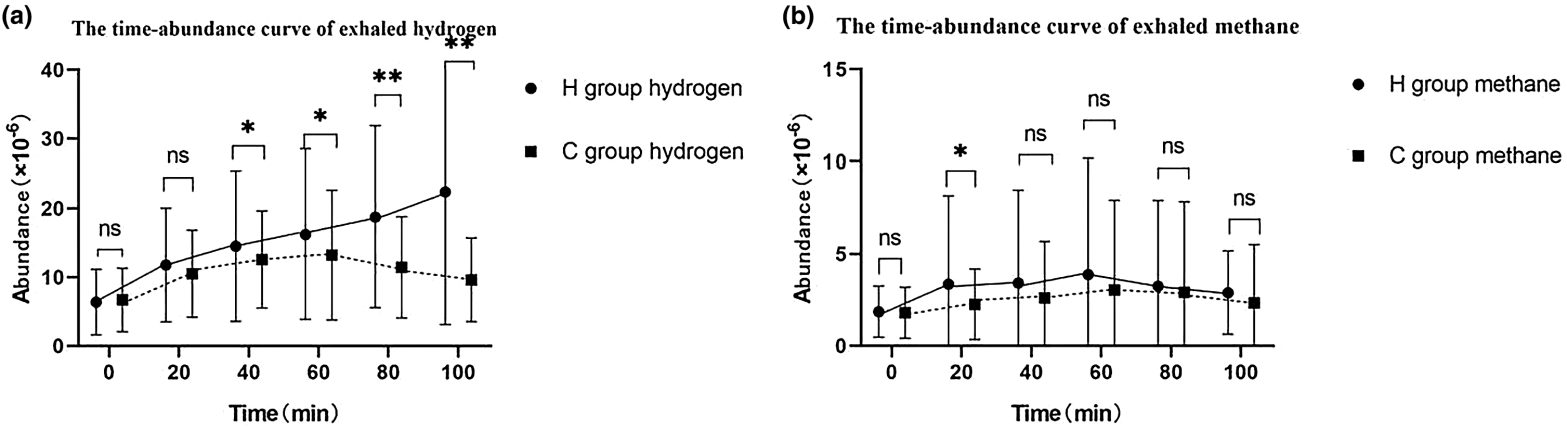
Hydrogen abundance versus methane abundance at various time points. (a) Comparison of hydrogen concentration between hypothyroidism in pregnancy group and control group at different time points. (b) Comparison of methane concentration between hypothyroidism in pregnancy group and control group at different time points.

As shown in Figure 1a, at the hydrogen 40, 60, 80, and 100 time points, the mean hydrogen abundance in the hypothyroidism in pregnancy group was higher than that in the normal pregnant women group, and the differences were statistically significant (*p* < .05). As shown in Figure 1b, the mean methane gas abundance in the hypothyroidism in pregnancy group was higher than that in the normal pregnant women group, and the difference in methane at time point 20 was statistically significant (*p* < .05).

**FIGURE 2:**
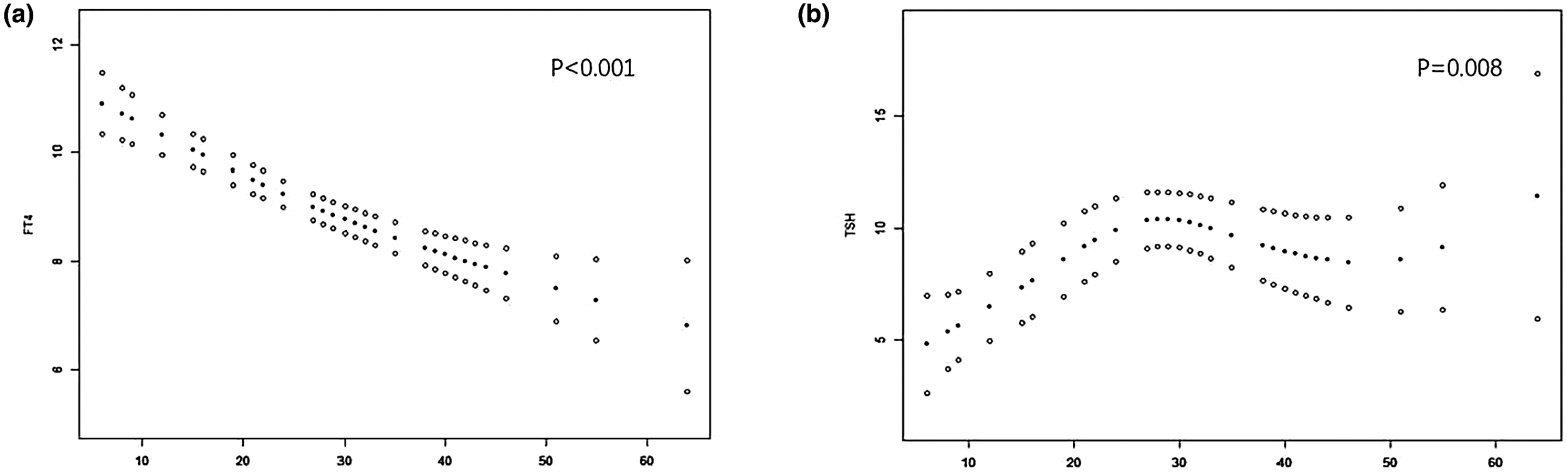
Correlation between hypothyroidism in pregnancy and SIBO. (a) Fitting of the highest value of hydrogen and methane in 90 min with the smooth curve of FT4 in patients with hypothyroidism during pregnancy. (b) The highest value of hydrogen and methane in 90 min in patients with hypothyroidism during pregnancy was fitted with the smooth curve of TSH. Where the error bar shows the 95% confidence interval.

Based on the SIBO diagnostic criteria, the highest value of hydrogen plus methane gas in 90 min was selected for smoothing curve fitting with FT4 and TSH in patients with hypothyroidism in pregnancy, and the results suggested that the highest value of hydrogen plus methane gas in 90 min was significantly negatively correlated with FT4 (*p* < .001, SD = 0.169) (Figure 2a), and the highest value of hydrogen plus methane gas in 90 min was not linearly correlated with TSH (*p* = .008, SD = 0.506) (Figure 2b). The SIBO conversion rates in the treated hypothyroidism in pregnancy combined with the SIBO group and normal pregnant women combined with the SIBO group were 71.4% and 64.3%, respectively.

### 3.3 Clinical symptoms

As shown in Table 2, (1) In terms of abdominal distension, there were significantly more in the hypothyroidism in pregnancy combined with SIBO group than in the hypothyroidism in pregnancy without SIBO group (*p* = .036), and there were significantly more in the normal pregnant women combined with SIBO group than in the normal pregnant women without SIBO group (*p* = .025). (2) In terms of diarrhea, the difference between the hypothyroidism in pregnancy combined with SIBO group and the hypothyroidism in pregnancy without SIBO group was not statistically significant (*p* = 1.000). The difference between the normal pregnant women combined with SIBO group and the normal pregnant women without SIBO group was not statistically significant (*p* = 1.000). (3) In terms of severe constipation, the difference between the hypothyroidism in pregnancy combined with SIBO group and the hypothyroidism in pregnancy without SIBO group was not statistically significant (*p* = .320). The difference between the normal pregnant women combined with SIBO group and the normal pregnant women without SIBO group was not statistically significant (*p* = .181).

**TABLE 2:** Comparison of clinical symptoms.

	Abdominal distension (%)	Diarrhea (%)	Severe constipation (%)
H‐S group (*n* = 28)	46.4	10.7	35.7
H‐N group (*n* = 22)	18.2	9.1	22.7
*p*	.036^a^ ^,^ *	1.000^b^	.320^a^
C‐S group (*n* = 14)	28.6	7.1	35.7
C‐N group (*n* = 36)	5.6	5.6	13.9
*p*	.025^b^ ^,^ *	1.000^c^	.181^b^

*Note*: H‐S group refers to hypothyroidism in pregnancy combined with SIBO group, H‐N group refers to hypothyroidism in pregnancy without SIBO group, C‐S group refers to normal pregnant women with combined SIBO group, C‐N group refers to normal pregnant women without combined SIBO group.

^a^ Represents the use of the χ^2^ test.

^b^ Represents the use of the corrected chi‐square test.

^c^ Represents the use of Fisher's exact probability test.

* In a row indicate that the medians of the different groups are significantly different (*p* < .05).

After 21 days of treatment with *Combined Bifidobacterium*, *Lactobacillus*, *Enterococcus*, *and Bacillus cereus Tablets*, *Live* combined with *levothyroxine sodium tablets*, in the group of hypothyroidism in pregnancy combined with SIBO, the conversion rate was 69.2% for bloating, 33.3% for diarrhea and 70.0% for severe constipation. After 21 days of treatment with *levothyroxine sodium tablets*, the conversion rate in the hypothyroidism in pregnancy without SIBO group was 75.0% for bloating, 0.0% for diarrhea, and 80.0% for severe constipation. After 21 days, in the group of normal pregnant women with SIBO, the rate of conversion of bloating was 75.0%, the rate of conversion of diarrhea was 100.0%, and the rate of conversion of severe constipation was 60.0%.

### 3.4 Serological test results

As shown in Table 3. Before treatment, compared with the hypothyroidism in pregnancy without SIBO group, FT4 was significantly lower in the hypothyroidism in pregnancy combined with SIBO group (*p* < .001), TSH and CRP were significantly higher (*p* = .001, *p* = .012), and there was no statistically significant difference in the percentages of TPOAb, WBC, neutrophils, and neutrophils percentage (*p* = .709, *p* = .252, *p* = .759, *p* = .835).

**TABLE 3:** Comparison of serological test results.

	*n*	FT4	TSH	TPOAb	WBC	Neutrophils	Neutrophils percentage	CRP
H‐S group	28	8.086 ± 1.071	10.001 ± 4.400	37.379 ± 9.596	8.710 ± 2.062	6.522 ± 1.993	68.777 ± 12.915	12.834 ± 9.420
H‐S group after treatment	28	10.246 ± 0.709	6.097 ± 2.012	29.236 ± 5.144	9.553 ± 1.887	7.494 ± 1.827	61.368 ± 13.588	8.203 ± 2.118
*t*		−10.68	4.057	3.647	−1.693	−1.958	1.874	2.508
*p*		<.001*	<.001*	.001*	.102	.061	.072	.018*
H‐N group	22	10.435 ± 1.186	6.530 ± 2.665	38.373 ± 8.926	9.405 ± 2.162	6.695 ± 1.911	67.914 ± 16.304	7.663 ± 3.886
H‐N group after treatment	22	10.343 ± 0.747	9.370 ± 3.870	29.250 ± 5.570	9.039 ± 2.427	7.270 ± 3.282	55.897 ± 16.670	11.421 ± 4.939
*t*		0.436	−2.49	4.52	0.491	−0.742	2.196	−2.616
*p*		.667	.021*	<.001*	.628	.467	.039*	.016*
*t* ^a^		−7.267	3.453	−0.375	−1.159	−0.309	0.209	2.634
*p* ^b^		<.001*	.001*	.709	.252	.759	.835	.012*

*Note*: H‐S group refers to hypothyroidism in pregnancy combined with SIBO group, H‐N group refers to hypothyroidism in pregnancy without SIBO group.

^a^ The *t*‐value of the H‐S group compared to the H‐N group.

^b^ The *p*‐value of the H‐S group compared to the H‐N group.

* In a row indicate that the medians of the different groups are significantly different (*p* < .05).

Compared with pre‐treatment, the treated hypothyroidism in pregnancy combined with SIBO group had significantly higher FT4 (*p* < .001), significantly lower TSH, TPOAb, and CRP (*p* < .001, *p* = .001, *p* = .018), and no statistically significant difference in the percentage of WBC, neutrophils, and neutrophils percentage (*p* = .102, *p* = .061, *p* = .072). The treated hypothyroidism in pregnancy without SIBO group showed significantly higher TSH and CRP (*p* = .021, *p* = .016), significantly lower TPOAb and neutrophils percentage (*p* < .001, *p* = .039), and no statistically significant difference in T4, WBC, and neutrophils (*p* = .667, *p* = .628, *p* = .467).

## 4 DISCUSSION

Existing research suggests that the thyroid is closely linked to gut flora (Kun et al., 2023). In our study, we found that the prevalence of SIBO increased significantly after having hypothyroidism in early pregnancy. Further smoothed curve fitting revealed that thyroid hormone levels in patients with hypothyroidism in pregnancy were significantly correlated with methane hydrogen exhalation. In addition, FT4 was lower and TSH was higher in hypothyroidism in pregnancy combined with SIBO than in hypothyroidism in pregnancy without SIBO, all of which suggest that hypothyroidism in pregnancy in early pregnancy is closely related to SIBO. The reasons why hypothyroidism during pregnancy can cause SIBO may be as follows: (1) hypothyroidism in pregnancy causes direct smooth muscle dysfunction, and the reduction of intestinal motility provides the conditions for SIBO (Ebert, 2010). (2) Thyroid hormone inhibits intestinal motility by reducing 5‐hydroxytryptamine, indirectly causing SIBO (Wang et al., 2021).

The mean exhaled hydrogen abundance and methane gas abundance in the group of pregnant women with hypothyroidism in pregnancy in this study were higher than those of normal pregnant women. A previous study by our group found that *Gammaproteobacteria* and *Provotella* were increased in the intestinal flora of hypothyroid patients in pregnancy compared to normal pregnant women (Wang et al., 2021), and these two bacteria were positively correlated with the amount of hydrogen production (Leite et al., 2020), which we hypothesize may be the reason for the higher mean hydrogen abundance in the hypothyroid group in pregnancy compared to the normal group.

The patients with SIBO are rich in colonic‐type bacteria, including gram‐negative aerobic and anaerobic bacteria, which can ferment carbohydrates into gas, thereby causing abdominal distension (Pimentel et al., 2020). Our study also found that abdominal distension symptoms are more pronounced in pregnant hypothyroid patients with combined SIBO compared to those without combined SIBO, with the following possible reasons: (1) the disruption of the intestinal barrier affects the absorption of nutrients in the intestine, and insufficient energy can lead to a decrease in intestinal motility. (2) Increased production of methane gas, which amplifies neuronal activity through cholinergic pathways, leads to dysmotility of the small intestine (Park et al., 2017). (3) Altered thyroid hormones cause a decrease in intestinal motility, which in turn causes abdominal distension (Ebert, 2010).

In this study, we found that FT4 was significantly lower and CRP was significantly higher in the group with hypothyroidism in pregnancy combined with SIBO compared to the group without SIBO, suggesting that the mechanisms associated between hypothyroidism in early pregnancy and SIBO may involve alterations in small intestinal bacteria, inflammatory factors, etc. On the one hand, hypothyroidism‐induced alterations in intestinal function relocate intestinal bacteria in the small intestine (Kun et al., 2023). Our findings support this reasoning: as expected, FT4 levels are negatively correlated with SIBO. On the other hand, our results suggest higher CRP after hypothyroidism in pregnancy combined with SIBO, suggesting an association with inflammation. The majority of microorganisms in the small intestine are gram‐negative bacteria, and lipopolysaccharides are the main component of the outer membrane lobules of this bacterium (Sperandeo et al., 2019). Upon binding lipopolysaccharide, the Toll‐like receptor 4 interacts with the myeloid differentiation factor 88 (MyD88), which is a central node of the inflammatory pathway (Kim et al., 2019).

Some studies have shown that probiotics can improve intestinal flora disorders, affecting human metabolism, and immune functions (Akram et al., 2023; Gyawali et al., 2023). Our findings suggest that patients with hypothyroidism in early pregnancy combined with SIBO treated with probiotics had a higher rate of SIBO conversion and improvement of clinical symptoms such as abdominal distension. The reasons for this phenomenon may be as follows: (1) The increase of *Lactobacillus* and *Bifidobacterium* spp. in the intestine can reduce adverse metabolites in the intestine, thereby reducing the occurrence of SIBO (Talebi, Karimifar, Heidari, Mohammadi, Asbaghi, et al., 2020). (2) The short‐chain fatty acids (SCFA) produced by probiotic fermentation can improve the permeability of the intestinal mucosa and increase the frequency of intestinal peristalsis, thus improving symptoms such as abdominal distension (Nickles et al., 2021).

In addition, probiotics can influence the intestinal inflammatory response (Ben Bacha et al., 2021). Our results also suggest that thyroid hormone levels improved significantly and inflammatory factors decreased after probiotic treatment in patients with hypothyroidism combined with SIBO during pregnancy. The main mechanisms of action may include: (1) The SCFA produced by probiotic fermentation can inhibit deacetylase through G protein‐coupled receptors, thereby inhibiting systemic inflammatory response (McLoughlin et al., 2017). (2) Organic acids produced by *Bifidobacterium*, lactic acid produced by *Lactobacillus acidophilus*, and SCFA produced by *Enterococcus faecalis* may increase the formation of lysozyme and inhibit the growth of pathogens (Delitala et al., 2017; Jandhyala et al., 2015). (3) *Lactobacillus acidophilus* can significantly reduce β‐glucuronidase activity in the feces, thereby reducing the inactivation of thyroid hormones (Talebi, Karimifar, Heidari, Mohammadi, & Askari, 2020; Uccello et al., 2012).

Of course, this experiment has some limitations. On the one hand, the study results have some regional limitations. On the other hand, the sample size of this study is limited.

In conclusion, this study clarified the association between gestational hypothyroidism in early pregnancy and SIBO and found a better efficacy of probiotic intervention in early pregnancy, thus providing a new direction for the study of gestational hypothyroidism and SIBO.

## AUTHOR CONTRIBUTIONS

**Miao Zhang:** Conceptualization (supporting); funding acquisition (equal); project administration (supporting); writing – original draft (lead); writing – review and editing (lead). **Yajuan Xu:** Conceptualization (lead); project administration (lead); writing – review and editing (supporting). **Zongzong Sun:** Investigation (lead); methodology (supporting); resources (supporting). **Yanjie Ban:** Investigation (supporting); methodology (supporting); resources (supporting). **Shanshan Zhai:** Data curation (lead); supervision (lead); validation (supporting). **Wentao Wang:** Data curation (supporting); visualization (supporting). **Mengqi Wang:** Data curation (supporting); visualization (supporting). **Jie You:** Formal analysis (lead); validation (lead). **Dongsun Chen:** Investigation (supporting); resources (supporting). **Shuanghui Zhu:** Investigation (supporting); resources (supporting). **Hui Guo:** Investigation (supporting); resources (supporting).

## FUNDING INFORMATION

This work was supported by Henan Provincial Science and Technology Research Project, LHGJ20210452 (MZ).

## CONFLICT OF INTEREST STATEMENT

The authors declare no conflicts of interest.

## ETHICS STATEMENT

The studies involving human participants were reviewed and approved by the Ethics Committee of the Third Affiliated Hospital of Zhengzhou University. Ethics: #2022‐254‐01. The patients/participants provided their written informed consent to participate in this study.

## CLINICAL TRIAL REGISTRATION NUMBER

ChiCTR1900026326.

## Data Availability

The original data is saved in https://figshare.com/s/9e2d2d918081b0c01c32, DOI: https://doi.org/10.6084/m9.figshare.14604087.
